# Post surgery circulating free tumor DNA is a predictive biomarker for relapse of lung cancer

**DOI:** 10.1002/cam4.980

**Published:** 2017-04-05

**Authors:** Wenwei Hu, Yang Yang, Longzhen Zhang, Jianxin Yin, Jingwei Huang, Lei Huang, Hua Gu, Gening Jiang, Jianmin Fang

**Affiliations:** ^1^ School of Life Sciences and TechnologyTongji UniversityShanghaiChina; ^2^ Shanghai Pulmonary HospitalShanghaiChina; ^3^ Tongji University Suzhou InstituteSuzhouJiangsuChina; ^4^ Collaborative Innovation Center for BiotherapyWest China HospitalSichuan UniversityChengduSichuanChina

**Keywords:** Circulating cell‐free DNA, lung cancer, relapse, surgery

## Abstract

Cancer cells release DNA fragments into plasma as circulating free DNA (cfDNA). However, quantitative measurement of tumor‐derived DNA in cfDNA remains challenge. The purpose of this study was to quantitatively assess tumor‐derived DNA in lung cancer patients. By optimizing competitive allele‐specific TaqMan PCR (CAST‐PCR), we assessed the copy number of mutated Kirsten rat sarcoma viral oncogene homolog (KRAS) and epidermal growth factor receptor (EGFR) alleles in the pre/post surgery plasma of 168 lung cancer patients. An absolute quantitative PCR method was developed to assess the number of total cfDNA. All mutations detected in tumors were also found in the plasma after surgery. At the time of 30 days after surgery, EGFR mutation of circulating cell‐free DNA was detected only in two patients who recurred in 4 months after surgery. Compared to that of normal control at 30 days after surgery, five patients who recurred in 4 months had significantly higher circulating cell‐free DNA (*P* < 0.001), whereas six patients who recurred after 4 months (*P* = 0.207) and five patients without recurrence (*P* = 0.901) demonstrated significantly lower circulating cell‐free DNA. Our findings suggest that cfDNA analysis in plasma is an alternative and supplement to tissue analysis and hold promise for clinical application. Stratification of patients according to cfDNA levels at 30 days after surgery might be helpful in selecting lung cancer patients for adjuvant therapy after surgery.

## Introduction

Cancer of the lung is the leading cause of death from cancer in the world, and accounts for 27% of all cancer‐related deaths in American 1. Surgery treatment is the first choice for patients with early stage lung cancer; and 5‐year survival rates of them range between 60% and 80% after surgical treatment, followed by adjuvant therapy 2. However, recurrence is common, and there are no clinically useful biomarkers to predict the risk of recurrence. Stratification of patients according to prognostic risk might be helpful in selecting lung cancer patients for adjuvant therapy 3. It is therefore urgently important to identify biomarkers to optimize therapeutic strategies.

Targeted therapy is one of the major treatments for cancer, but small proportion of patients can benefit from targeted therapies for the inevitable occurrence of resistance 4. Some resistance can be detected from the tissue sample. But for most patients, these detections could not be done for its invasive characteristic, and it always fail to reflect current tumor dynamics and drug sensitivity during therapy. Liquid biopsies offer the opportunity to investigate the molecular profiling of primary and metastasis tumors through simple and noninvasive blood tests 5, 6, 7. Moreover, the analysis of circulating tumor DNA (ctDNA) and circulating cell‐free DNA (cfDNA) in liquid biopsy provides the changes to study the “tumor dormancy” phenomenon 8, 9. Furthermore, dynamic monitoring tumor‐free nucleic acid copy number can assess the progress of the disease during and after treatment 10. However, the tumor‐free copy number of nucleic acids at different sampling time may vary, it is thus critical to determine the right time points for blood sampling.

Competitive allele‐specific TaqMan PCR (CAST‐PCR) is a highly specific and sensitive technology that can detect rare amounts of mutated DNA in a large background of normal wild‐type genomic DNA. In CAST‐PCR, an allele‐specific primer and FAM dye‐labeled MGB (Minor Groove Binder) probe detects mutant allele, whereas an MGB oligonucleotide blocker suppresses the wild‐type allele. In particular, the sensibility for TaqMan Mutation Detection Assays (TMDA) has been shown to be at least 0.5% for frequent mutations 11, 12, 13. Thus, we hypothesized that detecting the KRAS and EGFR mutation of cfDNA in liquid biopsies by CAST‐PCR may be applied to monitor the disease progression of lung cancer during therapy.

In this study, we assessed the copy number KRAS and EGFR mutation alleles by CAST‐PCR in plasma. We further investigated the dynamic levels of KRAS and EGFR mutations in plasma collected from lung cancer patients during surgery, and analyzed the quantitative relationship of KRAS and EGFR mutations in the plasma with cfDNA, as well as the correlation of cfDNA levels with the recurrence of lung cancer after surgery.

## Patients and Methods

### Patient materials

A prospective study was conducted at the Shanghai Pulmonary Hospital and School of Life Sciences and Technology of Tongji University to identify predictive and prognostic biomarkers during the surgical treatment for lung cancer. Inclusion criteria were histopathologically verified lung cancer. Cancer tissue samples from primary tumors and blood samples during treatment were collected for marker analysis. Informed consents were obtained from all patients.

### Sample collection and DNA purification

Tumor tissues were histologically evaluated to confirm the number of tumor cells, which were subjected to a proteinase K (Sigma‐Aldrich, St. Louis, MO) digestion at 56°C overnight. Genomic DNA was extracted with standard isopropanol precipitation.

A 9‐mL peripheral blood sample was collected in Heparin Sodium tubes from each patient at baseline. After collection, plasma was obtained by centrifugation at 2000*g* for 10 min within 2 h, and stored at −80°C. Total DNA isolated from 1.2 mL of plasma using a Tiangen Serum/Plasma Circulating DNA Kit (Tiangen Biotech, Beijing, China), according with manual.

### KRAS and EGFR mutational analysis

To obtain unlimited amounts of positive control materials for KRAS and EGFR assays, a site‐directed PCR mutagenesis was used to obtain eight plasmids, containing one mutation in EGFR (c.2573T>G p.L858R) and seven mutations in KRAS (c.34G>T p.G12C, c.34G>A p.G12S, c.34G>C p.G12R, c.35G>T p.G12V, c.35G>A p.G12D,c.35G>C p.G12A and c.37G>A p.G13S). Eight plasmid solutions were prepared from five aliquots of the linearized stock plasmid. Dilutions were made in water to yield a range spanning 5^0–12^ copies/*μ*L. Each plasmid that carried a KRAS or EGFR mutation was used to generate a standard curve. Analysis of KRAS and EGFR mutation in primary tumor and metastases was conducted using a CAST‐PCR kit (Applied Biosystems, Foster City, CA). Water controls and wild‐type donor DNA controls were used as negative controls. Site‐directed mutated plasmids were used as positive controls. Automatic threshold and baseline determination was used in this study. The integrity of DNA was determined by electrophoresis with 4% agarose gel.

Each PCR reaction mixture was composed of 10 *μ*L of Taqman Genotyping Master Mix (2x), 2 *μ*L of CAST‐PCR Allele‐1 assay (10x), 8 *μ*L of DNA template. Thermal cycling conditions were: 95°C for 10 min, 5 cycles of 92°C of l5 sec and 58°C of 1 min), followed by 45 cycles of 92°C of 15 sec and 60°C of 1 min. Data during 45 cycles of amplification were collected and analyzed using the sequence detection system software (SDS, Applied Biosystems, Foster City, CA). An absolute quantification value was obtained by comparing the amplification cycle threshold of the DNA control sample to the CT of the target samples. Each plasma sample was tested with independent quantification assays at least three times.”

### Quantification of cfDNA, and KRAS and EGFR in plasma

Standard curves were generated by serial dilutions of KRAS and EGFR site‐directed mutated plasmids in 5‐fold decrements into 100 ng normal donor DNA (pool of DNA purified from normal blood samples). The specificity of the different in‐house assays was tested in 100 ng normal donor DNA as a reference; and from the standard curves, these were calculated to 0.025% for KRAS c.37G>A, 0.004% for KRAS c.34G>A, and better than 0.001% for KRAS c.34G>T, c.34G>A, c.34G>C, c.35G>T, c.35G>A and c.35G>C and EGFR c.2573T>G.

Quantification of cfDNA was calculated by the copy number of gCYC with equation Copy number (gCYC)= 10^{y intercept (gCYC) ‐meanCt (gCYC)}/ slope (gCYC)}^ 14. The mutated KRAS copy number was calculated with the equation copy number (KRAS)= 5^{y intercept (KRAS) ‐meanCt (KRAS)/slope (KRAS)}^. A similar method was used to quantify EGFR mutations.

### Statistical analysis

The correlation between cfDNA and plasma mutated KRAS/EGFR (pmKRAS/ pmEGFR) alleles were investigated with Spearman's rank correlation. A multivariate Cox regression analysis was conducted using a backward stepwise elimination process, which eliminates the predictor with the largest *P* value in each step until all predictors in the final model had *P *<* *0.2. Two‐sided *P* values were considered significant when *P *<* *0.05 (No correction for multiple testing was applied). Statistics were conducted in SPSS v. 20.0.

## Results

### Patient characteristics

A cohort of 168 patients with verified primary lung cancer was recruited in this study from May 2012 to November 2012. Baseline clinical and pathophysiological characteristics of patients are listed in Table 1. Median follow‐up was 21 months, which ended by September 2014. Eleven patients had recurrence by the end of the 21th month; wherein, recurrence occurred in five patients 4 months after surgery and recurrence occurred in the other patients after 4 months.

**Table 1:** Patients characters

	ADC	SCC	LCLC	SCLC	AdCa	ND	Tatol	Controls
No. of patients	86 (51.2%)	62 (36.9%)	5 (3%)	2 (1.2%)	2 (1.2%)	11 (6.5%)	168	10
Age, years								
Median (range)	59 (26–77)	62 (39–80)	66 (59–79)	53 (41–65)	53 (51–55)	61 (27–76)		58
Gender								
Female	49	3	0	1	1	2	50 (32%)	4
Male	37	59	5	1	1	9	106 (68%)	6
TNM stage								
I	44	19	1	1	2	3	70	
II	6	22	1	1	0	3	33	
III	25	15	3	0	0	2	45	
IV	6	3	0	0	0	0	9	
V	0	0	0	0	0	0	0	
ND	5	3	0	0	0	3	11	
T stage								
1	6	3	0	0	0	0	9	
2	65	40	2	1	2	6	116	
3	4	14	1	1	0	1	21	
4	5	3	2	0	0	1	11	
ND	6	2	0	0	0	3	11	
N stage								
0	49	37	3	0	2	7	98	
1	5	9	0	2	0	0	16	
2	25	14	2	0	0	1	42	
3	0	0	0	0	0	0	0	
4	0	0	0	0	0	0	0	
X	1	0	0	0	0	0	1	
ND	6	2	0	0	0	3	11	
Mutation								
EGFR p.L858R	21	8	3	2	1	1	36	
KRAS p.G12C	2	1	0	1	2	1	7	
KRAS p.G12D	1	1	0	0	0	0	2	
KRAS p.G12V	2	1	0	0	0	1	4	
KRAS p.G13S	1	1	0	0	0	1	3	
Recurrence								
4M	3	2	0	0	0	0	5	
12M	3	1	0	0	0	0	4	
18M	2	0	0	0	0	0	2	
ND							157	

Histological classification was determined according to World Health Organization classification of tumors, pathology and genetics of tumors of the lung, pleura, thymus, and heart. 2004.

LCLC, large cell lung cancer; ADC, lung adenocarcinoma; SCC, squamous cell carcinoma of the lung; SCLC, small cell lung cancer; AdCa, adenosquamous; Carcinoma; Controls, Healthy persons.

### Correlation of KRAS and EGFR mutations in tumor tissue with plasma

All these samples were assessable for comparative analysis of KRAS and EGFR mutations of tissues to that of peripheral blood (Table 2). Thirty‐six EGFR and sixteen KRAS mutations were found in tumor tissues of 168 patients. Fifteen KRAS mutations detected in tumor tissues were also found in peripheral blood (15/16, 94%). All EGFR L858R mutations detected in tumor tissues were found in peripheral blood (100%). These data have shown that most EGFR and KRAS mutations in lung cancers could be detected in patient plasma.

**Table 2:** Comparison of (A) KRAS 2EXON^a^ and (B) EGFR L858R^b^ detection in tissue and plasma

(A)			
	Tumor KRAS mutation	Tumor KRAS wild type	Total
Plasma KRAS mutation	15	0	15
Plasma KRAS wild type	1	152	153
Total	16	152	168

(B)			
	Tumor EGFR mutation	Tumor EGFR wild type	Total
Plasma EGFR mutation	36	0	36
Plasma EGFR wild type	0	132	132
Total	36	132	168

a Sensitivity, 99.4%; specificity, 100%; positive predictive value, 100%; negative predictive value, 94%.

b Sensitivity, 100%; specificity, 100%; positive predictive value, 100%; negative predictive value, 100%. EGFR, epidermal growth factor receptor.

Next, the levels of KRAS and EGFR mutations in the plasma were determined in the 52 patients (36 with EFGR L858R mutation and 16 with KRAS exon 2 mutation) with early stage lung cancer before surgery (day zero). KRAS mutation fragment ranged from five to 25 mutant copies per sample (median, 12 mutant copies per sample). EGFR mutation fragment ranged from five to 49 mutant copies per sample (median, 36 mutant copies per sample).

### The levels of EGFR mutations to assess tumor dynamics

**Figure 1:**
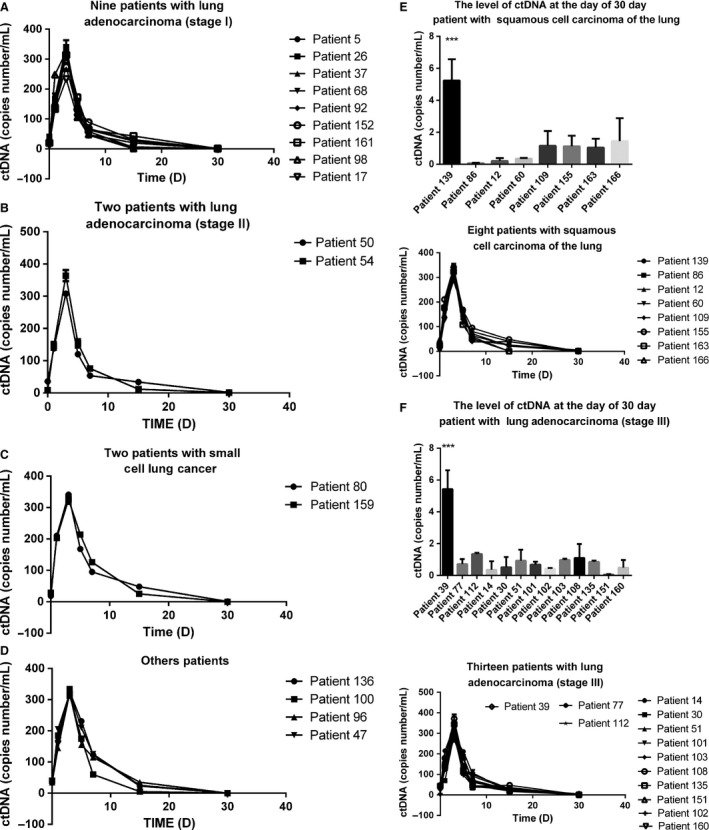
Dynamic alteration of circulating mutant DNA levels in plasma of early lung cancer patients during surgical treatment. (A) Nine patients with lung adenocarcinoma (stage I); (B) two patients with lung adenocarcinoma (stage II); (C) two patients with small cell lung cancer; (D) other patients; (E) eight patients with squamous cell lung carcinoma: patient 139 recurred in 4 months, patient 86 recurred in 9 months, and the other patient had no recurrence; (F) 13 patients with lung adenocarcinoma (stage III): patient 39 recurred in 4 months after surgery, whereas patient 77 and 112 recurred 4 months after surgery.

To evaluate the correlation of circulating mutant DNA levels with tumor dynamics, we analyzed the mutant DNA of 36 patients with EGFR L858R mutation at different time points following surgery. A sharp increase in the levels of EGFR mutation in the plasma at 24 h after surgery was shown in all patients with incomplete resections, with a median of 163 copies per sample (range: 124–251; Figs. 1, A–D). The levels of EGFR mutation in the plasma reached a maximum median of 336 copies per sample (range: 242–390) at 3 days after surgery, but rapidly dropped at the day of discharge (3–7 days after surgery) in all subjects and became undetectable in 34 cases at 30 days after surgery. Interestingly, EGFR mutation in the plasma remained detectable in two patients (patient 39 and 139) at 30 days after surgery, who had recurrence 4 months after surgery. At 30 days, EGFR mutation in the plasma levels in patient 39 (Fig. 1E) and patient 139 (Fig. 1F) were significantly higher than the other patients. This result showed the levels of EFGR mutations may be biomarker of prognosis of surgery treatments.

### Correction between KRAS and EGFR mutation in the plasma and cfDNA levels in plasma

**Figure 2:**
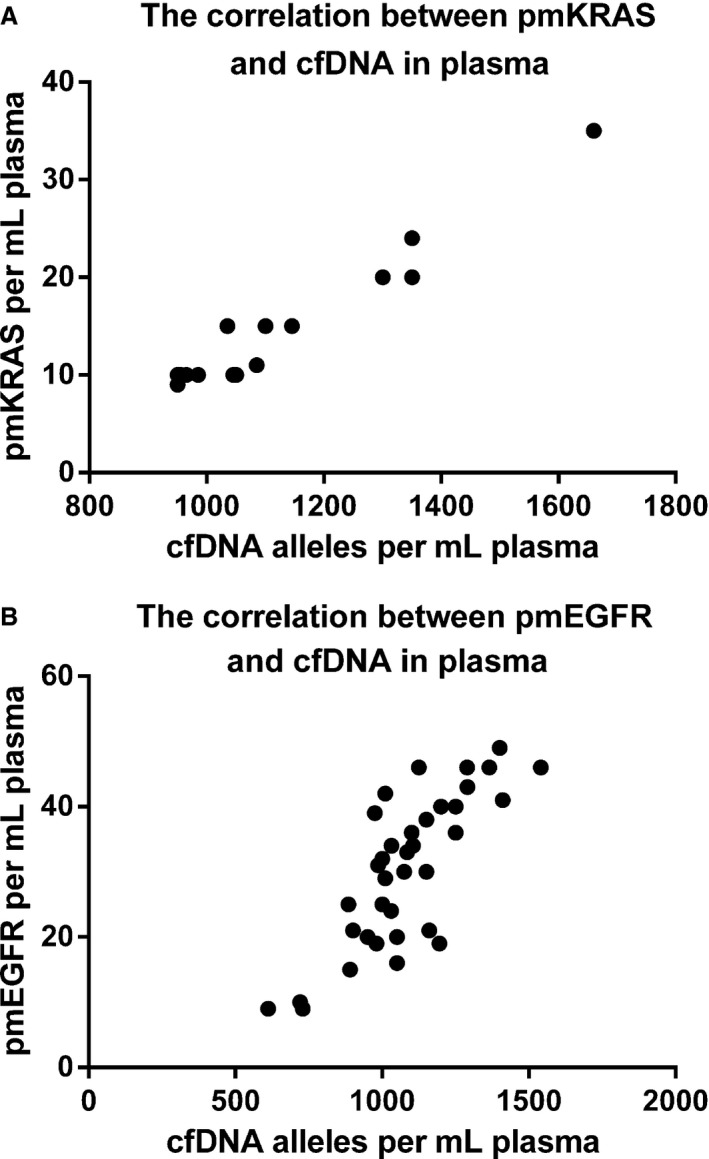
Correlation between KRAS/EGFR mutation and cfDNA levels in plasma. (A) A significant correlation between KRAS mutation and cfDNA levels was observed in plasma (Spearman's rank correlation was 0.8996; *P *=* *0.0001). Median level of cfDNA was 1117 alleles per mL of plasma (range: 950–1660). There was no significant difference in cfDNA levels between patients with KRAS mutation (median: 1017; range: 950–1660) and wild‐type disease (median: 1043; range: 628–2623; *P *>* *0.05). Median level of pmKRAS was 11 (range: 9–35). (B) A significant correlation between EGFR mutation and cfDNA level was found in plasma (Spearman's rank correlation was 0.7339; *P *=* *0.0009). Median level of cfDNA was 1062.5 alleles per mL of plasma (range: 612–1540). There was no significant difference in cfDNA levels between patients with EGFR mutation (median: 1062.5; 612–1540) and wild‐type disease (median: 1043; range: 828–2623; *P *>* *0.05). The median level of pmKRAS was 31.5 (range: 9–49). cfDNA, circulating cell‐free DNA; KRAS, Kirsten rat sarcoma viral oncogene homolog.

To assess the correlation of KRAS and EGFR mutations with cfDNA in plasma, we plotted KRAS or EGFR mutated alleles per mL of plasma against the number of cfDNA alleles per mL of plasma. As shown in Figure 2A, there was a clear correlation between the level of KRAS mutations and cfDNA in plasma (Spearman's rank correlation was 0.8996, *P *<* *0.0001). Similarly, a clear correlation between the level of EGFR mutations and cfDNA in plasma (Spearman's rank correlation was 0.7339, *P *<* *0.0009) was obtained (Fig. 2B).

### Circulating cell‐free DNA for assessing tumor dynamics

To assess the correlation of cfDNA levels with tumor dynamics, plasma DNA levels in all 168 lung cancer patients were determined during the follow‐up period. The trend of the levels of cfDNA in plasma is very similar to the levels of EGFR mutation in plasma.

**Figure 3:**
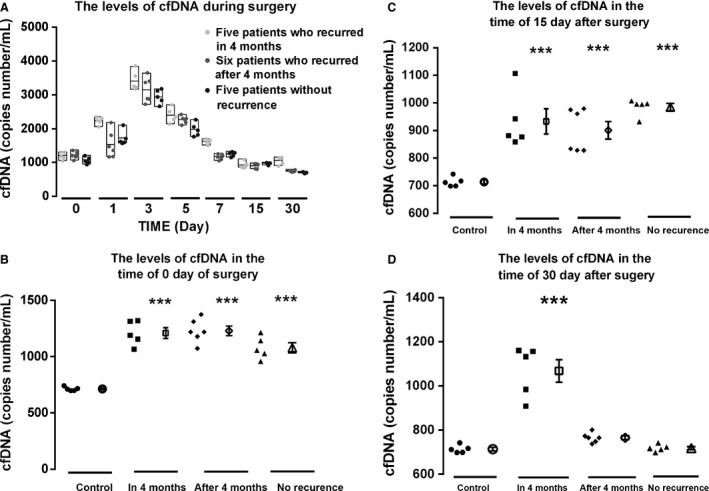
Dynamic alteration of cfDNA levels in the plasma (A) Dynamic alteration of cfDNA levels in the plasma of early lung cancer patients with and without recurrence during surgery treatment. (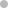) Five patients who had no recurrence; (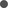) Six patients who recurred after 4 months; ● Five patients who recurred in 4 months. (B) The cfDNA levels at day zero of surgery; (C) cfDNA levels at day 15 after surgery; (D) the cfDNA levels at the day 30 after surgery. Five patients recurred in 4 months; 6 patients recurred after 4 months; five patients without recurrence and five normal health controls. cfDNA, circulating cell‐free DNA.

The levels of cfDNA in relapse‐free patients’ plasma decreased progressively reaching similar levels to that of healthy humans, and remained low at 30 days of follow‐up (mean: 1072; range: 984–1106; Fig. 3A). On the contrary, in patients with relapse within 4 months after surgery, plasma DNA levels decreased following primary surgery; but levels remained as the time of 15 days (Fig. 3A). Moreover plasma DNA levels of patients after 4 months were similar to that of relapse‐free patients (Figs. 3A) (Wilcoxon rank sum test, *P *=* *0.05 and *P *<* *0.001 at four and 10 months, respectively). These data suggest the levels of cfDNA at the time 30 days after surgery have value for making predictions of subsequent relapse in 4 months.

### Prognostic value of cfDNA levels

Eleven patients had recurrence after surgery: five patients in 4 months and six patients after 4 months. We compared the cfDNA levels of patients with recurrence in 4 months and after 4 months with that of five normal persons and five patients who had no relapse at the time of before surgery (day zero), 15 days and 30 days after surgery. At day zero, all patients had significantly higher levels of cfDNA than that in normal control patients (*P* < 0.001, Fig. 3B). At day 15, cfDNA levels remained decreased in all patients, but were still significantly higher than healthy controls (*P* < 0.001, Fig. 3C). In sharp contrast, at day 30, cfDNA levels of patients with recurrence after 4 months and without recurrence decreased to that of normal controls; but cfDNA levels of patients with recurrence within 4 months remained significantly high (*P *<* *0.001, Fig. 3D). There were no significant differences in cfDNA levels between normal controls and patients with recurrence after 4 months (*P *=* *0.207) or without recurrence (*P *=* *0.901). These results revealed that patients with recurrence in 4 months had high cfDNA levels, but patients with recurrence after 4 months and patients without recurrence had the same cfDNA levels as controls at 30 days after surgery; while at 15 days, all the patients who have been treated with surgery had significantly higher cfDNA levels than normal controls.

Quantitative levels of KRAS mutation in the plasma were not entered into the model, because the sample size for mutant patients was limited. However, an isolated multivariate analysis including pmKRAS and pmEGFR levels grouped as quartiles has shown that high levels of cfDNA or/and KRAS and EGFR mutation in the plasma at 30 days were both strong predictors of poor outcome in 4 months. This result suggests an independent prognostic value of cfDNA, consistent with the strong correlation of KRAS and EGFR mutation in the plasma with cfDNA levels (Fig. 2).

### The levels of cfDNA in plasma and tumor type

**Figure 4:**
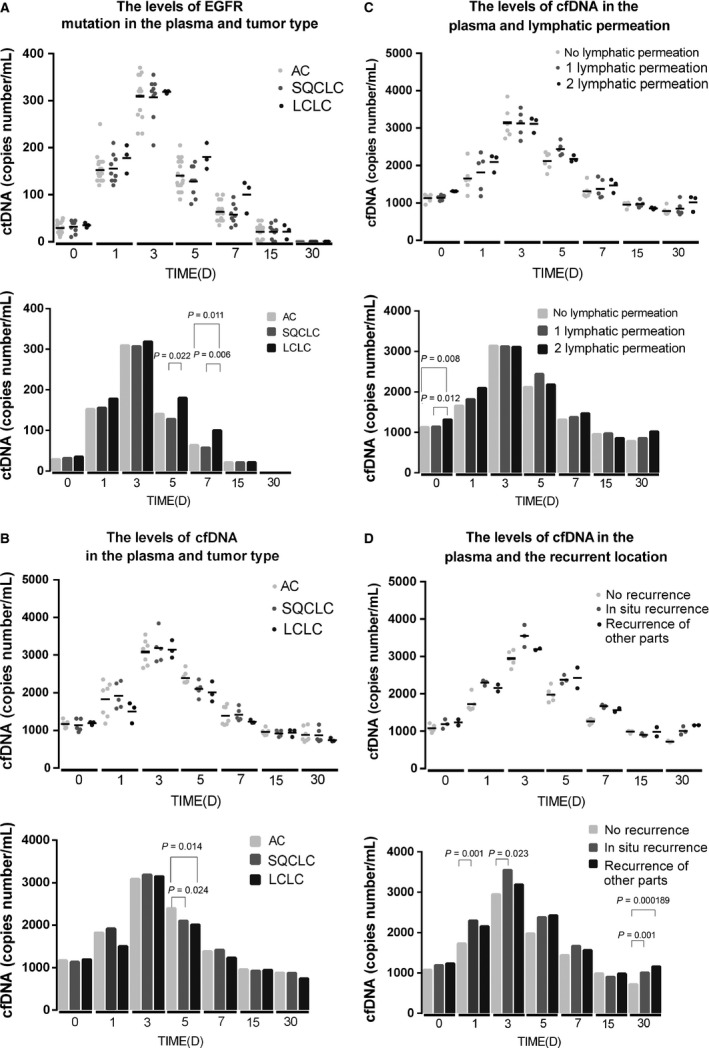
The levels of cfDNA in cancer types, *lymphatic permeation and recurrence location*. (A) The analysis of the levels of EGFR mutation DNA in plasma revealed that significant difference in the time of 5 day (EGFR mutation DNA in plasma in the time of 5 day: 128.3333 copies/mL and 180 copies/mL in the squamous cell carcinoma, and large cell lung cancer group, respectively; *P* = 0.022) and 7 day (EGFR mutation DNA in plasma in the time of 7 day: 63.6842, 57.7778 and 100 in the squamous cell carcinoma, and large cell lung cancer group, respectively; P1/3 = 0.011, P2/3 = 0.006). (B) The cfDNA in plasma copies/mL in the time of 7 day after surgery: 2396.63, 2101.36, and 2011.37 in lung adenocarcinoma, the squamous cell carcinoma, and large cell lung cancer group, respectively; P1/2 = 0.024, P1/3 = 0.014. (C) The analysis of the levels of cfDNA in plasma revealed that significant difference before surgery (cfDNA in plasma copies/mL before surgery: 1129.235, 1138.814, and 1314.91 in no lymphatic permeation, 1 lymphatic permeation and 2 lymphatic permeation group, respectively; P1/3 = 0.008, P2/3 = 0.012). (D) The analysis of the levels of cfDNA in plasma revealed that significant difference between no recurrence and in situ recurrence in the time of 1 day (cfDNA in plasma copies/ml at the time of 1 day after surgery: 1729.14, 2297.42, *P* = 0.001), 3 day (cfDNA in plasma copies/mL at the time of 3 day after surgery: 2944.12, 3548.48, *P* = 0.023) and 30 day (cfDNA in plasma copies/mL no recurrence, in situ recurrence and recurrence of other parts at the time of 30 day after surgery: 718.47 (1), 1008.32 (2), and 1158.40 (3), p1/2 = 0.001, p1/3 = 0.000189). cfDNA, circulating cell‐free DNA.

Integrity analysis revealed a significant difference in the time of 5 day (EGFR mutation DNA in plasma in the time of 5 day: 128.3333 copies/ml and 180 copies/mL in the squamous cell carcinoma, and large cell lung cancer group, respectively; *P* = 0.022; Fig. 4A) and 7 day (EGFR mutation DNA in plasma in the time of 7 day: 63.6842, 57.7778 and 100 in the squamous cell carcinoma, and large cell lung cancer group, respectively; *P* = 0.011, *P* = 0.006; Fig. 4A). The levels of cfDNA in plasma showed significant difference in the time of 7 day (cfDNA in plasma copies/ml in the time of 7 day after surgery: 2396.63, 2101.36, and 2011.37 in lung adenocarcinoma, the squamous cell carcinoma, and large cell lung cancer group, respectively; P1/2 = 0.024, P1/3 = 0.014; Fig. 4B). Because postoperative chemotherapy began in 30 days after the surgery, the influence of different chemotherapy on the levels of cfDNA had not been taken into account in this study.

### The levels of cfDNA in plasma and lymphatic permeation/ recurrence location

The cfDNAs in plasma before surgery in no lymphatic permeation, 1 lymphatic permeation and 2 lymphatic permeation groups were 1129.235, 1138.814, and 1314.91 copies/mL, respectively (P1/3 = 0.008, P2/3 = 0.012; Fig. 4C). However, this difference was not found after surgery. Moreover, a significant correlation the recurrent location and cfDNA levels were found after surgery (Fig. 4D). The cfDNA in plasma no recurrence and in situ recurrence groups were 1729.14 and 2297.42 copies/ml (*P* = 0.001) at 1 day after surgery, 2944.12 and 3548.48 copies/ml (*P* = 0.023) at 3 day after surgery.

## Discussion

In this study, we found that the levels of KRAS or EGFR mutations in plasma were quite different from patient to patient before surgery, but most EGFR and KRAS mutations in lung cancers could be detected in patient plasma; and the recurrence rates between patients with and without detectable KRAS and EGFR mutation in the plasma at 30 days after surgery were significant. Moreover, KRAS and EGFR mutations were positively correlated with cfDNA levels in plasma; and cfDNA levels at the time of surgery and at 30 days after surgery were predictive for subsequent relapse in 4 months. Importantly, high levels of cfDNA and KRAS and EGFR mutation in the plasma at 30 days were both strong predictors of poor outcome in 4 months. Our results indicate that the levels of KRAS or EGFR mutation in plasma are positively correlated with cfDNA in plasma, and the selection of different blood sampling time points is particularly important, suggesting the independent diagnostic and prognostic value of EGFR and KRAS mutation in the plasma of early lung cancer patients.

Surgery provides the best chance for lung cancer patients, and standard of care for early stage lung cancer patients is lobar resection 15, 16. However, almost 20% of these patients are not suitable for surgery treatment due to severe cardiac and/or respiratory comorbidities 17, 18. For these patients, targeted therapy is another choice, but response depends on the identification of the mutated target gene^19^, ^20^. Since solid tumor tissues are impossible to obtain, plasma test is the best choice for these patients 21, 22, 23. Due to the heterogeneity and chromosome instability of cancer cells, targeted therapy may be compromised 24, 25. Indeed, Lee et al. found a relation of early tumor recurrence and chromosomal instability with drug resistance 26, 27, 28. Misale et al. performed genotyping on cfDNA exacted from plasma of 24 patients with colorectal cancer, and showed that clinical resistance arose through the acquisition of mutations detected by cfDNA 22, 29. Similarly, EGFR‐activating mutations in cfDNA were found to be present before initiating erlotinib therapy, but decreased or disappeared during therapy 30, 31. Mutations subsequently reemerged along with drug resistance mutations after continued treatment with erlotinib in patients with EGFR‐mutant lung cancer 32. The KRAS or EGFR mutations in the blood analysis may help overcome some of the obvious limitations of tissue analysis for KRAS and EGFR mutations 30, 31. Moreover, previous studies found that cfDNA levels of mutated target genes are lower in patients in the early stage than that in patients in the advance stage 28, 32. Our findings suggest that EGFR and KRAS mutations in the plasma can also be detected by CAST‐PCR in patients even in the early stages.

It has been shown that patients with low KRAS allele at baseline may benefit from targeted therapies 21; but at present, these patients are not considered candidates for EGFR inhibitor treatment, because they harbor KRAS mutant disease 33, 34, 35. We therefore suggest the use of the levels of KRAS or EGFR mutations in the blood as a supplement to tissue KRAS and EGFR mutation analysis for selection prior to treatment 36, 37, 38, 39. Although all early lung cancer patients with mutations have been detected, the copy numbers varied among different individuals in our study. Taking into account the different types of cancer can cause different levels of free nucleic acid, adenocarcinoma, squamous cell carcinoma, and large cell lung cancer groups were established to detect EGFR mutation DNA and cfDNA in plasma (Fig. 4 A and B). The results indicate that different cancer types can affect the levels of plasma cfDNA originated from tumor cells and cfDNA, and this difference may be in the short term after surgery. These results suggest that the levels of KRAS or EGFR mutations in the plasma as a supplement to tissue KRAS and EGFR mutation analysis.

Recurrence after surgery is common, and there are no clinically useful biomarkers to predict the risk of recurrence in lung cancer 40, 41. Detecting cfDNA and EGFR and KRAS mutations in the plasma may help clinicians to assess the survival probability and select precise treatment plan 6, 42. However, the optimum time of blood sampling remains unclear for patients with lung cancer 43, 44, 45. Continuous monitoring of cfDNA and EGFR and KRAS mutations in the plasma levels in lung cancer patients after operations may determine the optimum time of blood sampling 46, 47, 48, 49. In this study, we found that EGFR and KRAS mutation in the plasma levels reached its highest levels on the 3rd day after surgery, and gradually decreased thereafter; but there was no significant difference in cfDNA and KRAS and EGFR mutation in the plasma levels among blood samples collected before and at 1, 3, 5, 7, and 15 days after surgery. Interestingly, cfDNA and KRAS and EGFR mutation in the plasma levels in blood samples collected 30 days after surgery were significantly different between the group that had relapse in 4 months and the other groups. Our results suggest that the levels of mutation and cfDNA may be useful biomarkers for lung cancer patients after surgery treatment and 30 days after surgery may be an important time for collecting blood samples to detect.

The cfDNA in plasma from patients with cancer originates from normal nonmalignant cells, as well as necrotic and apoptotic tumor cells; but neither the origin nor the fate of the circulating DNA is fully understood 39, 50, 51, 52. A previous study has shown a correlation between quantitative cfDNA levels and tumor‐specific KRAS and EGFR mutations in plasma, which triggered us to hypothesize that increasing levels of cfDNA in patients with cancer are primarily of tumor origin 44, 53, 54, 55, 56. To test this hypothesis, we detected EGFR and KRAS mutations, as well as cfDNA levels in plasma; and explored the potential value of the quantification of mutated alleles in the clinical setting during surgery. In general, patients with high levels of KRAS and EGFR mutation in the plasma 30 days after surgery had poor prognosis compared with the subgroup of patients harboring KRAS and EGFR mutations at low levels. The data we have present also indicate that it may not only be the KRAS and EGFR status itself, but rather the quantitative amount of cfDNA derived from tumor that influences the disease behavior. In addition, we found that different lymphatic permeation can affect the levels of cfDNA before surgery, and this difference disappeared after surgery. A significant correlation was observed between in situ recurrence and cfDNA levels in the time of 1 day and 3 day after surgery. Therefore, stratification of patients according to cfDNA levels might be helpful in selecting lung cancer patients for adjuvant therapy after surgery.

In conclusion, KRAS and EGFR mutations can be detected in peripheral blood as an alternative and supplement to tissue analysis; and quantitative levels of cfDNA, pmKRAS, and pmEGFR are associated to the clinical outcome of the surgical treatment of lung cancer. Quantification of cfDNA, as well as KRAS and EGFR mutations, in the plasma is a great potential clinical tool for clinicians to do pretreatment testing and design precise treatment plan.

## Ethical approval

All procedures performed in studies involving human participants were in accordance with the ethical standards of the institutional and/or national research committee and with the 1964 Helsinki declaration and its later amendments or comparable ethical standards. This article does not contain any studies with animals performed by any of the authors.

## Conflict of Interest

We declare that we do not have any commercial or associative interest that represents a conflict of interest in connection with the work submitted.
